# Supplementary material for: Antioxidant Efficacy of Green-Synthesized Silver Nanoparticles Promotes Wound Healing in Mice
Source: Pharmaceutics. 2023 May 17;15(5):1517. doi: 10.3390/pharmaceutics15051517 (PMC10222969; doi:10.3390/pharmaceutics15051517)
Supplement: Supplementary file 1 [file pharmaceutics-15-01517-s001.zip › pharmaceutics-2329947-supplementary.pdf]

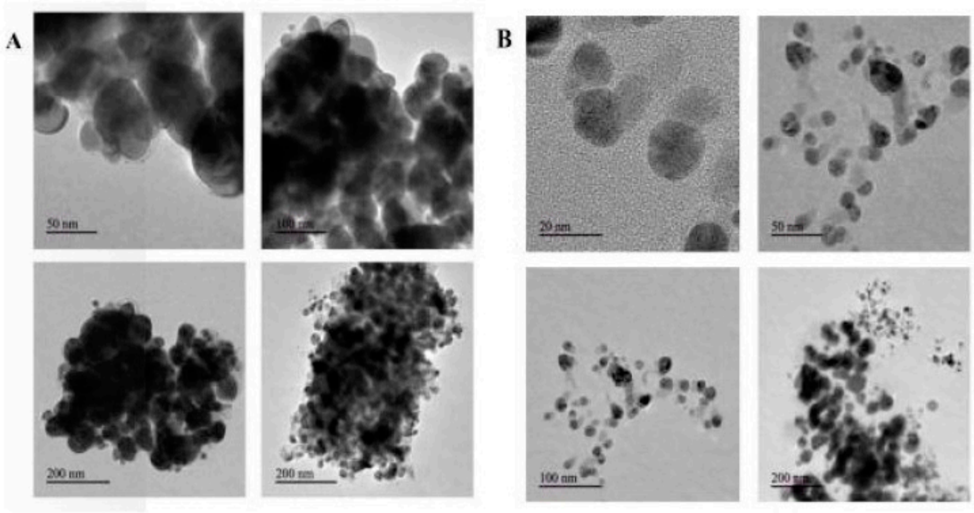

Figure S1. TEM images of biosynthesized silver nanoparticles from (**A**) *Catharanthus roseus* and (**B**) *Azadirachta indica* plant extracts [34].

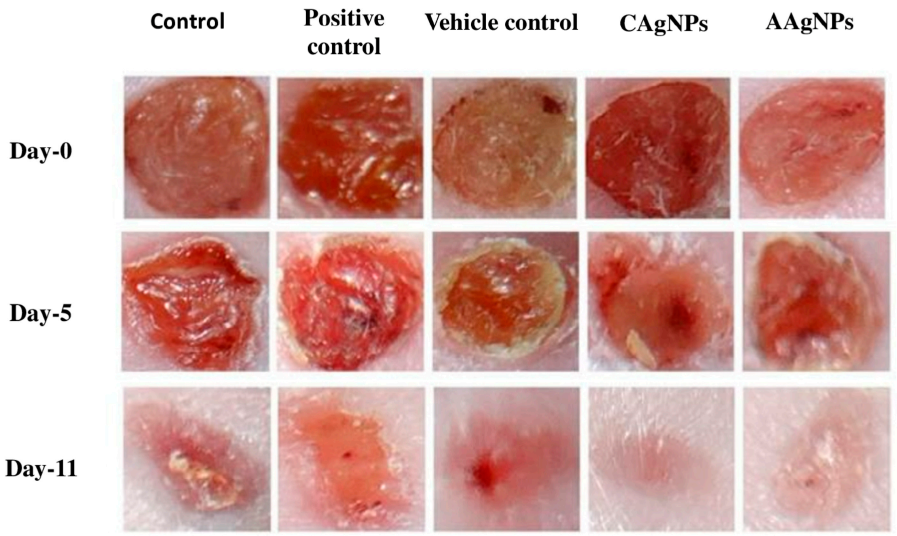

(**a**)

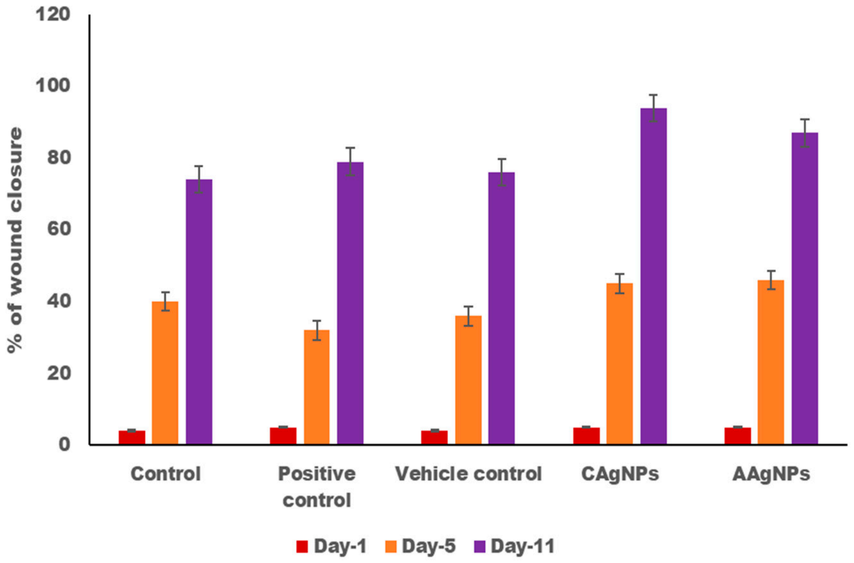

(**b**)

Figure S2. (**a**): Representative photographs showing wound closure after treatment with green-synthesized silver nanoparticles of *Catharanthus roseus* and *Azadirachta indica.* (**b**): Graphical representation of percent of wound closure after treatment with green-synthesized silver nanoparticles of *Catharanthus roseus* and *Azadirachta indica.*
